# Supplementary material for: Restoration of MHC-I on Tumor Cells by Fhit Transfection Promotes Immune Rejection and Acts as an Individualized Immunotherapeutic Vaccine
Source: Cancers (Basel). 2020 Jun 12;12(6):1563. doi: 10.3390/cancers12061563 (PMC7352176; doi:10.3390/cancers12061563)
Supplement: Supplementary file 1 [file cancers-12-01563-s001.pdf]

Supplementary Materials

Restoration of MHC-I on Tumor Cells by Fhit Transfection Promotes Immune Rejection and Acts as an Individualized Immunotherapeutic Vaccine

María Pulido, Virginia Chamorro, Irene Romero, Ignacio Algarra, Alba S-Montalvo, Antonia Collado, Federico Garrido and Angel M. Garcia-Lora

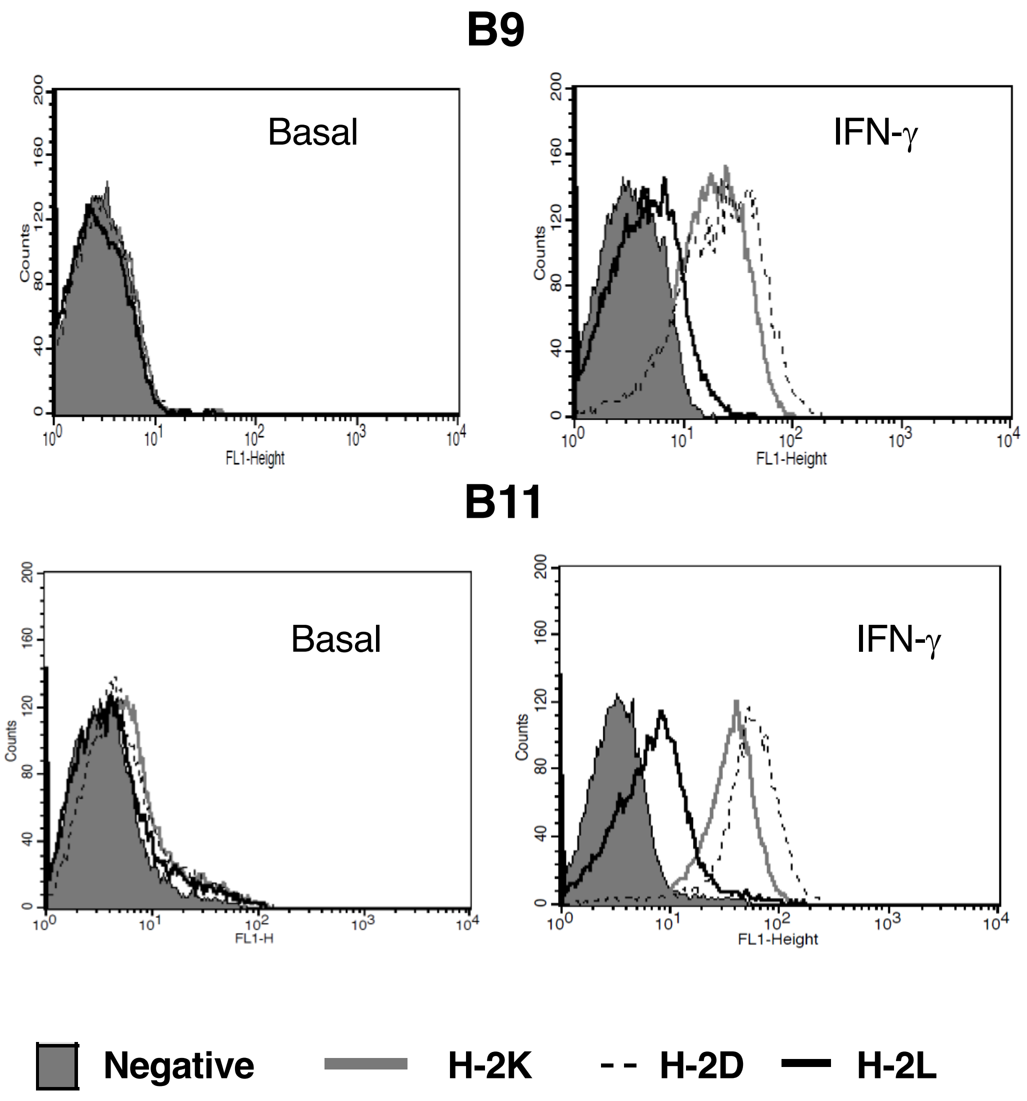

Figure S1. MHC-I phenotypes of B9 and B11 tumor cell lines under baseline conditions and after treatment with IFN-γ: H-2 Kd (gray line), H-2 Dd (dotted line), and H-2 Ld (black line). The two cell lines show negative MHC-I expression, and treatment with IFN-γ induced the expression of all three H-2 molecules.

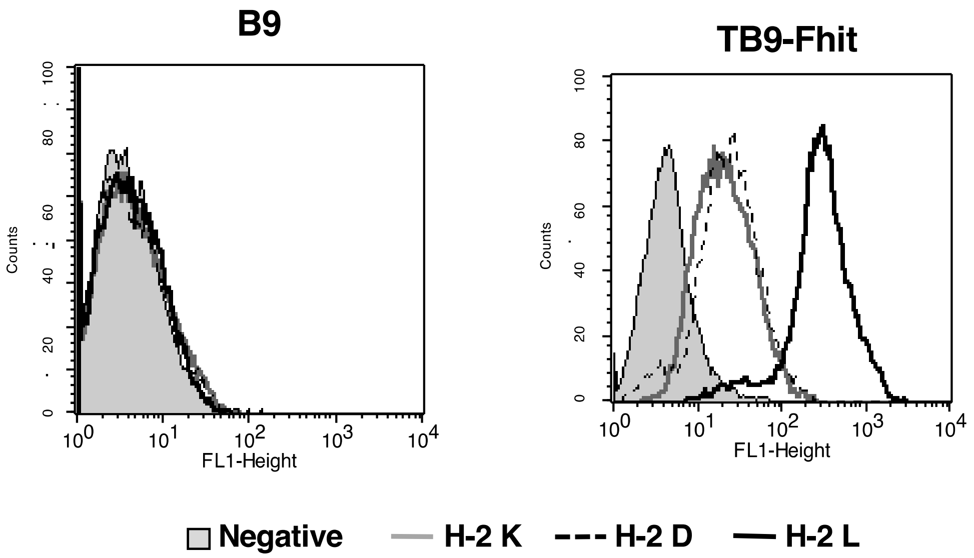

Figure S2. MHC-I phenotypes of B9 and TB9-Fhit tumor cell lines under baseline conditions: H-2 Kd (gray line), H-2 Dd (dotted line), and H-2 Ld (black line). Fhit transfection induced expression of all three H-2 molecules on MHC-I negative B9 tumor cells.

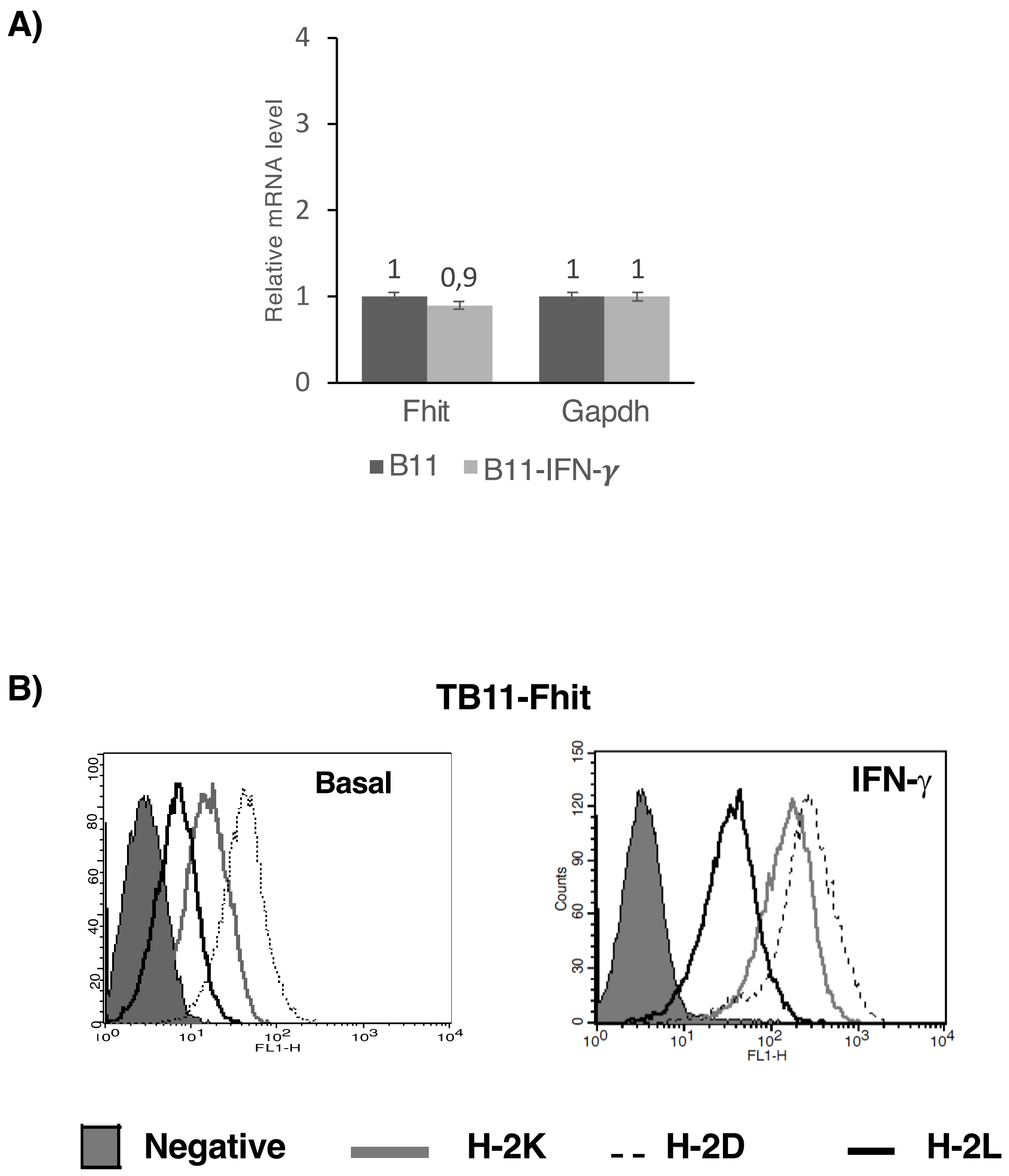

Figure 3. (**A**) The Fhit gene does not change its expression at the transcriptional level after IFN-! treatment of B11 tumor cells. (**B**) MHC-I phenotypes of TB11-Fhit tumor cell line under baseline conditions and after treatment with IFN-γ: H-2 Kd (gray line), H-2 Dd (dotted line), and H-2 Ld (black line). TB11-Fhit tumor cells show positive expression of three H-2 class I molecules, and all molecules were induced after treatment with IFN-γ.

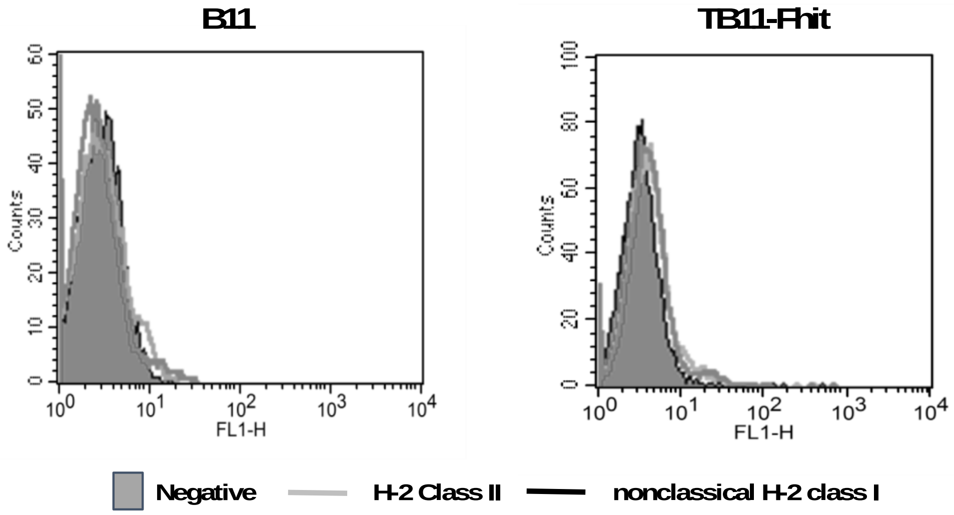

.

Figure S4. Nonclassical MHC-I and MHC-II phenotypes of B11 and TB11-Fhit tumor cell lines under baseline conditions: H-2 class II (gray line), and nonclassical H-2 class I (black line). Both cell lines show negative expression.

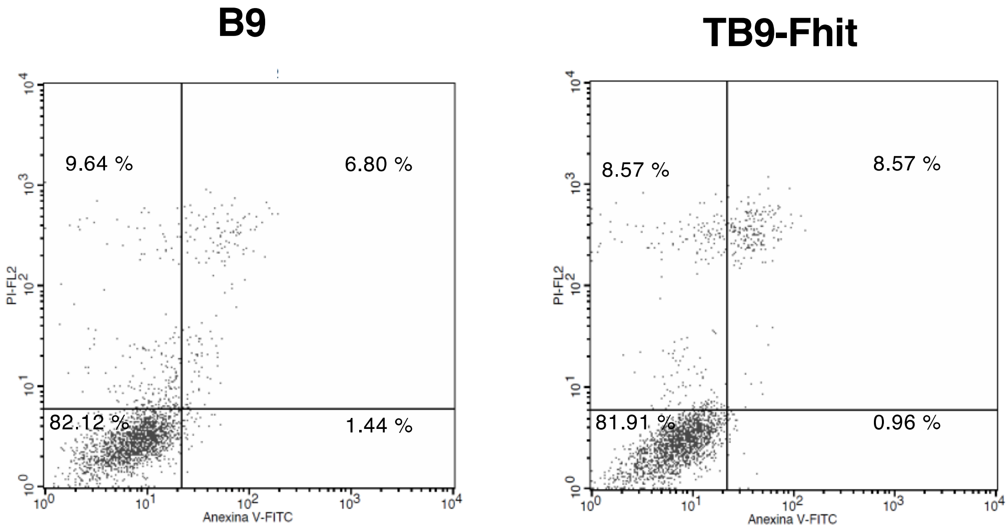

Figure S5. Apoptosis and necrosis data in B9 and TB9-Fhit tumor cell lines. Total apoptotic and necrotic cells, characterized with Annexin-VFITC labeling, were estimated by flow cytometry; 10.000 events were analysed for each sample. Cells in the lower left quadrant are viable, those in the lower right quadrant are apoptotic and those in the upper left and upper right are late apoptotic/necrotic. No significant differences were found.

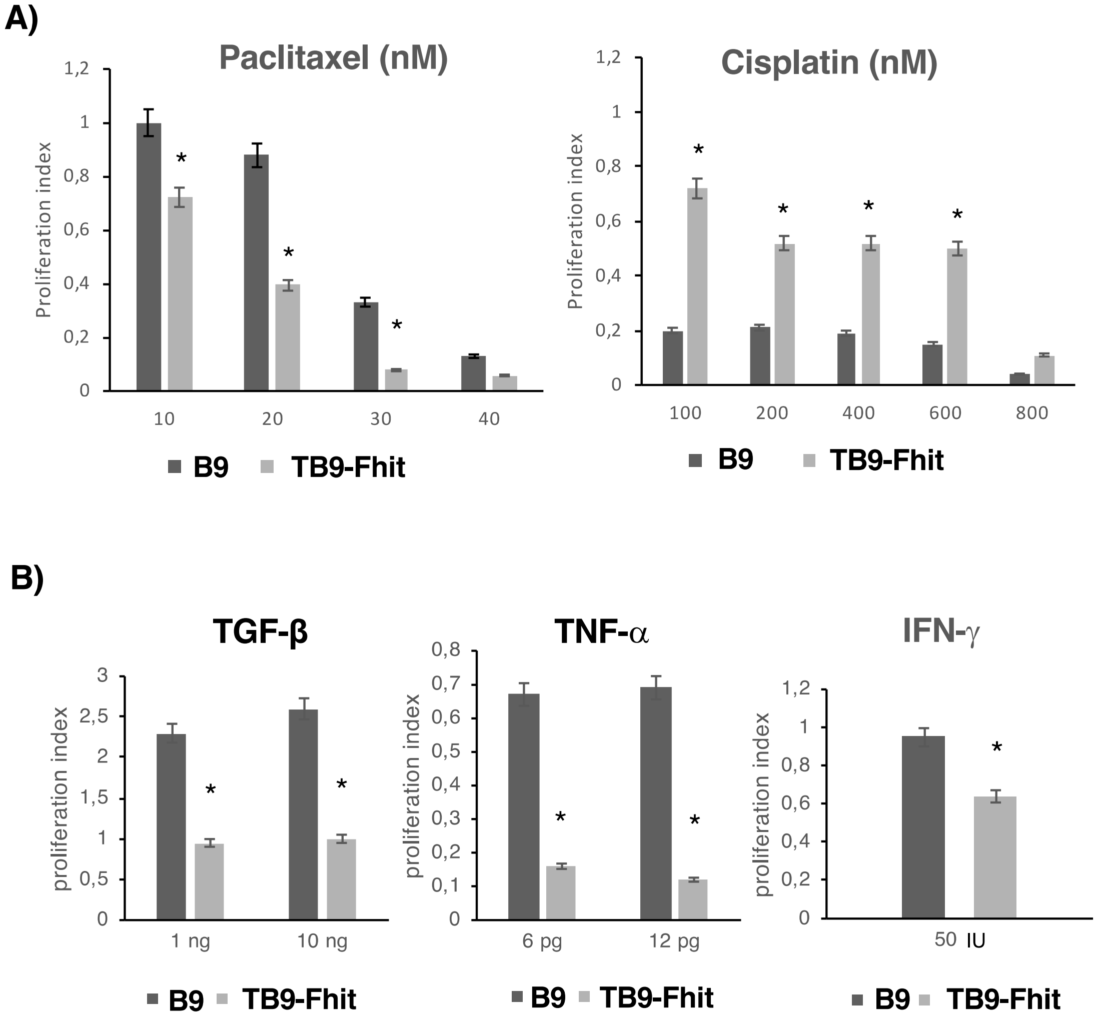

Figure S6. (**A**) In vitro susceptibility of B9 and TB9-Fhit to paclitaxel and cisplatin chemotherapeutic agents. Fhit transfection produced greater susceptibility to paclitaxel and lesser susceptibility to cisplatin. (**B**) In vitro susceptibility of B9 and TB9-Fhit to TGF-b, TNFa and IFN-g. TGF-b increased the proliferative rate of non-transfected tumor cells but had no effect on this rate in Fhit-transfected tumor cells. Fhit transfection increased the susceptibility to TNF-a and IFN-g. The Proliferation index was calculated as the ratio between treated and nontreated cells. Values are depicted as means ± SD of three independent experiments performed in duplicate. * $p < 0.01$. A two-tailed Student's t-test was used for statistical analysis.

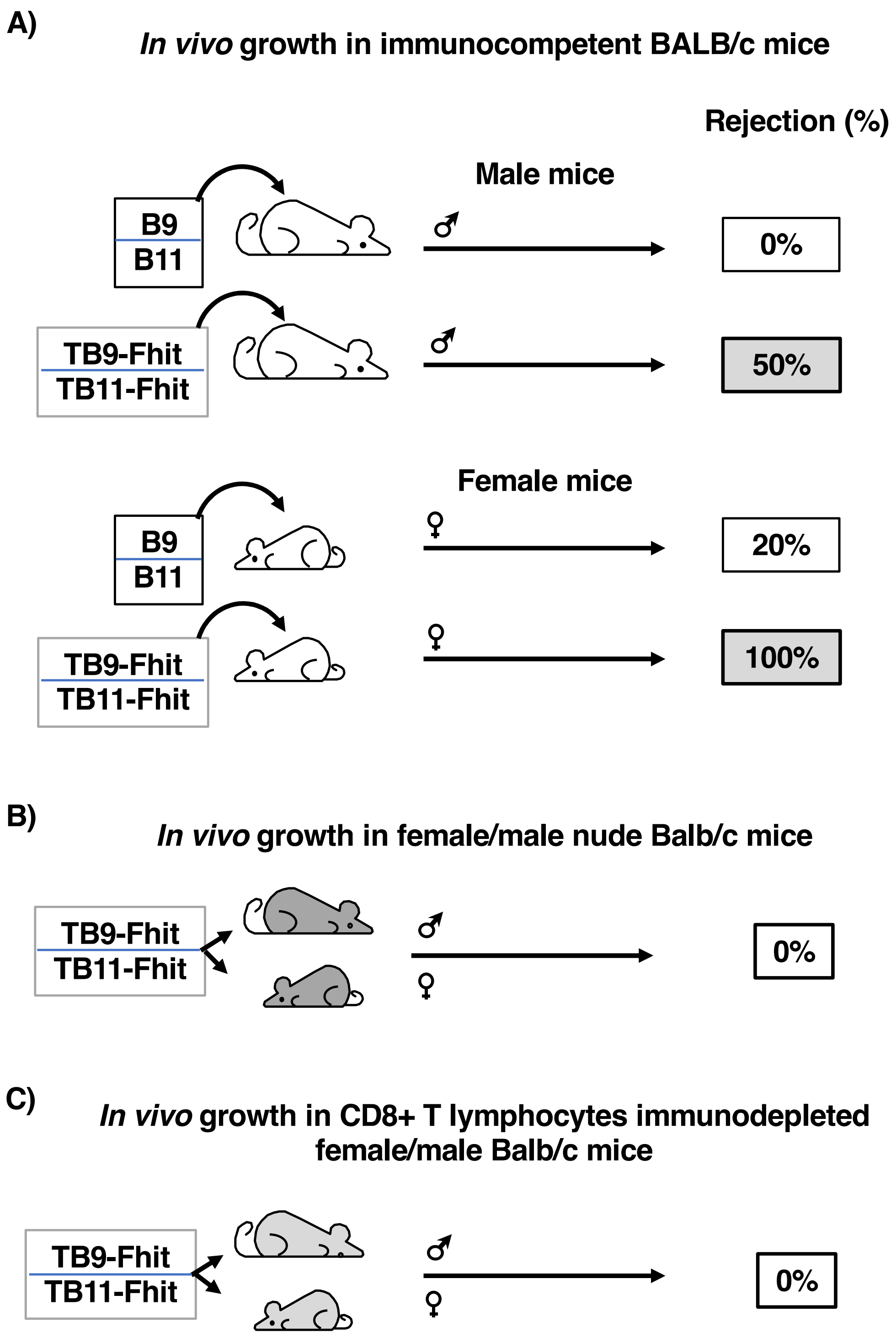

Figure S7. (**A**), In vivo growth of B9, B11, TB9-Fhit, and TB11-Fhit tumor cells in female/male immunocompetent mice. TB11-Fhit was rejected in 100% of female mice and 50% of male mice. (**B**) In vivo growth of TB9-Fhit and TB11-Fhit tumor cells in female/male nude mice. The two tumor cells grew in all animals. (**C**) In vivo growth of TB9-Fhit and TB11- Fhit tumor cells in CD8+ T lymphocyte-immunodepleted male/female immunocompetent mice. The two tumor cells grew in all animals.

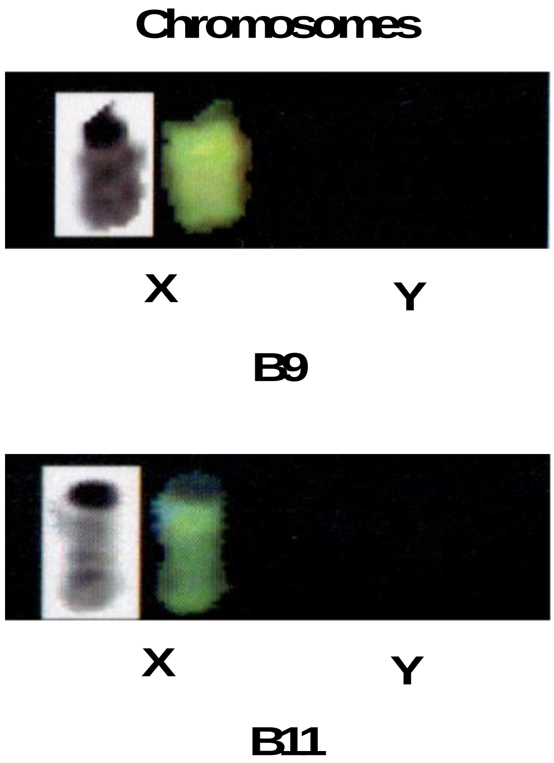

Figure S8. Spectral Kariotyping of the B9 and B11 tumor cell lines revealed that both cell lines are X chromosome monochromatic and lack a Y chromosome.

Table S1. Changes in activation markers in T lymphocyte subpopulations.

**CD3⁺ CD8⁺ Lymphocytes[a]**				
**Females**	**Control**	**14 dpi[b]**	**21 dpi[b]**	**28 dpi[b]**
**CD45⁺ CD3⁺ CD8⁺ CD134⁺**	101 ± 17	134 ± 19**	121 ± 16**	140 ± 7**
**CD45⁺ CD3⁺ CD8⁺ CD154⁺**	58 ± 4	76 ± 7**	91 ± 14**	118 ± 18**
**CD45⁺ CD3⁺ CD8⁺ CD137⁺**	106 ± 10	134 ± 12**	128 ± 19**	120 ± 10**
**CD45⁺ CD3⁺ CD8⁺ CD69⁺**	265 ± 14	281 ± 14**	296 ± 24**	322 ± 26**
**CD45⁺ CD3⁺ CD8⁺ CD95⁺**	111 ± 21	76 ± 9**	134 ± 13**	143 ± 15**
**Males**	**Control**	**14 days**	**21 days**	**28 days**
**CD45⁺ CD3⁺ CD8⁺ CD134⁺**	118 ± 9	118 ± 15	125 ± 15	144 ± 17**
**CD45⁺ CD3⁺ CD8⁺ CD154⁺**	78 ± 6	81 ± 10	97 ± 9**	106 ± 9**
**CD45⁺ CD3⁺ CD8⁺ CD137⁺**	132 ± 10	122 ± 5**	115 ± 14**	119 ± 12**
**CD45⁺ CD3⁺ CD8⁺ CD69⁺**	237 ± 9	229 ± 8*	249 ± 27*	250 ± 9**
**CD45⁺ CD3⁺ CD8⁺ CD95⁺**	76 ± 8	61 ± 9	156 ± 12**	117 ± 12**
**CD3⁺ CD4⁺ lymphocytes[a]**				
**Females**	**Control**	**14 dpi[b]**	**21 dpi[b]**	**28 dpi[b]**
**CD45⁺ CD3⁺ CD4⁺ CD134⁺**	197 ± 22	292 ± 66**	176 ± 18**	234 ± 29**
**CD45⁺ CD3⁺ CD4⁺ CD154⁺**	97 ± 5	109 ± 15**	108 ± 33**	94 ± 21
**CD45⁺ CD3⁺ CD4⁺ CD137⁺**	104 ± 5	148 ± 29**	140 ± 16**	122 ± 19**
**CD45⁺ CD3⁺ CD4⁺ CD69⁺**	292±19	395 ± 131**	295 ± 54	334 ± 22**
**CD45⁺ CD3⁺ CD4⁺ CD95⁺**	106±16	91 ± 8**	146 ± 21**	140 ± 9**
**Males**	**Control**	**14 days**	**21 days**	**28 days**
**CD45⁺ CD3⁺ CD4⁺ CD134⁺**	189 ± 33	181 ± 42	179 ± 28	227 ± 42**
**CD45⁺ CD3⁺ CD4⁺ CD154⁺**	78 ± 19	65 ± 5**	45 ± 8**	74 ± 11
**CD45⁺ CD3⁺ CD4⁺ CD137⁺**	132 ± 16	99 ± 5**	80 ± 10**	117 ± 9**
**CD45⁺ CD3⁺ CD4⁺ CD69⁺**	277 ± 67	288 ± 29	201 ± 22**	328 ± 47**
**CD45⁺ CD3⁺ CD4⁺ CD95⁺**	149 ± 27	51 ± 11**	143 ± 19	200 ± 23**

[a] Data represent number of activated CD8⁺ or CD4⁺ T cells among 1x10⁴ CD45⁺ cells. [b] Days post injection (dpi) of the TB11-Fhit tumor cells. * $p < 0.05$ compared with control group. ** $p < 0.01$ compared with control group. (n = 8 mice/group; mean ± SEM).

Table S2. Primers for Real-time RT-PCR.

Gene Name	Primer Sequence (5′ → 3′) Fw	Rev	Product Lenght [bp]	Acc. No.
**APM**				
H2-Kd	CCATCCACTGTCTCCAACACG	CCACCTGTGTTCCTTCTCATC	113	XM_017318971.1
H2-Dd	GCCTCCTTCATCCACCAAGAC	CACAGCTCCAAGGATGACCAC	81	NM_010380.3
H2-Ld	CGTCCACTGACTCTTACATGG	CCACAGCTCCAATGATGGCC	75	NM_001267808.1
Tap1	GCTGTTCAGGTCCTGCTCTC	CACTGAGTGGAGAGCAAGGAG	106	NM_013683.2
Tap2	AGGAGCCTGTGCTGTTCTCG	CTATGAAGTCGTCTGCACAGG	116	NM_011530.3
LMP2	CCTCTGCACCAGCACATCTTC	CGTGTAGCTCCAGCTGGTAG	94	NM_013585.2
LMP7	GGACCTCAGTCCTGAAGAGG	CAACCGTCTTCCTTCATGTGG	117	NM_010724.2
Calnexin	GCAGCTGAAGAGCGTCCATGG	TCATCCTTCACATCTGGCTGG	155	NM_001110500.1
Calreticulin	AGCAGATGAAGGACAAGCAGG	CCTCTCATCTTCTTCGTCCTC	140	NM_007591.3
Tapasin	CAGCTACCTCCAGTCACTGC	CCTAGCACCTTGAGGAGTCC	194	NM_001025313.1
Fhit	GCAAGATGGTCCTGAAGCTGG	CCATCTCCTTCTCAGATCTCC	167	NM_001360141.1
Tfap2a	GTCACTAGTGGAAGGAGAAGC	AGCCAGCAGGTCAGTGAACTC	190	NM_001122948.2
GAPDH	TCAAGAAGGTGGTGAAGCAGG	GCATCGAAGGTGGAAGAGTGG	117	NM_001289726.1
β-Actin	CAACACAGTGCTGTCTGGTGG	CTCCTTCTGCATCCTGTCAGC	63	NM_007393.5
Anpep	TGAACCAACAGACAGCTGTCC	CAGTAGTGATCCTCCTGTCC	229	NM_008486.3
Blmh	TCACTGCTGTCTCAGAGAAGG	ACATGCTTCTTGTCCACCACC	160	NM_178645.4
Erap1	TCCTTCTCAAGTTGCTGAAGG	CTCCTGAGCATCCTCTCTGAG	82	NM_030711.5
Lap3	ATCAGAGCTGCTGTTGCAGC	ACCTTCTTCTTCTGCTTCAGG	152	NM_024434.6
Npepps	CTGGAGAAGGTCATCTAGACG	GACAGGACTCCTTAGGTCAGC	155	NM_008942.3
Pdi	GTTCACTGAACAGACAGCTCC	GTGTGTCTTGATCTCACCTCC	52	NM_011032.3
ERp57	TCATGCAGGAGGAGTTCTCG	TGCTACCACAACCTTGACAGG	140	NM_007952.2
LMP10	ACCTCAGCTCTACGAGGTGC	CTTCCACCAACAGCTCTTGC	152	NM_013640.3
PA28a	TGGTCACTACCTGGTTGCAGC	GTGTGAAGGTTGGTCATCAGC	106	NM_011189.1
Thop1	TCAGGACCTCCTGGAGAAGC	CTGTCTGTGTGTGCAGGACC	110	NM_022653.4
Tpp1	CCTCAGCAGCATCTACATCC	AGCTCCAGTGTCACCTGAGG	96	NM_009906.6
Tpp2	TGTCCAGGAGGAACTACATCC	ACCTCCTGGTGCACTGATGC	174	NM_009418.3
**IFN-γ pathway**				
Ifngr1	CTGTCCTAGAGAGTGAGACG	TCTTCCTGTTCTGCTGCTTCG	88	NM_010511.3
Ifngr2	AGAGCAACTCCATTGTGCTGG	ATCAGGATGACTTGCTGCAGC	175	NM_008338.4
Irf1	CAGACATCGAGGAAGTGAAGG	TCCACACAGCTTCCTCTTGG	158	NM_001159396.1
Irf2	TCAGCATGAGTGAGCTCTACC	TGTTGCTGAGGTACTGCTTGC	156	NM_008391.4
Irf9	GTTCCTGGAGCATCAACTTCC	ACTCCACCTGCTCCATGCTG	158	NM_001159418.1
Jak1	CAGTCTCTGTGCTGACCAGG	CACACTCAGGTTCTTGGAGTC	89	NM_146145.2
Jak2	CAGCAAGCATGATGAGTCAGC	CTCTCCACAGACACAGACACC	71	XM_011247155.2
Kpna1	GTACTAGCTGATGCTTGCTGG	ACTCCTGCATCGATGACTGC	83	NM_008465.5
Ptpsh1	ACTACGTGAAGAACCAGCTGC	ACGATGACACGAGTGTTCTCC	130	NM_013545.3
Ptpsh2	GACTGTGACATCGACGTTCC	TACTGTGCTTCTGTCTGGACC	80	NM_011202.3
Socs1	TCGAGCTGCTGGAGCACTAC	TCAGGTAGTCACGGAGTACC	171	NM_001271603.1
Socs3	GACCAAGAACCTACGCATCC	ACCAGCTTGAGTACACAGTCG	105	NM_007707.3
Stat1	ACAACATGCTGGTGACAGAGC	CTCAACACCTCTGAGAGCTGG	88	NM_001357627.1
Stat2	TGCAGCAGCAGAAGTCCTGC	TCCTTCAGCTGCTTCAGTAGC	127	NM_019963.2
Stat3	GATCGTGACTGAGGAGCTGC	GTTGGAGATCACCACAACTGG	109	NM_011486.5
Crm1	TGAGCTCTACAGAGAGAGTCC	TACAACCAGTACCTCCTCTGG	166	NM_134014.3
**Cell cycle**				
Cdc25a	CCTACTGATGGCAAGCGTGTC	CTTCAGGACATACAGCTCAGG	147	NM_007658.3
Cdc25b	CCATCATGCCTTGTAGCCTGG	AGTCGTTAGCTGCACGGTCC	120	NM_023117.4
Cdk2	GGCCAGGAGTTACTTCTATGC	GCTCCGTCCATCTTCATCCAG	104	NM_183417.3
Cdk3	CCAGATGCCTGACTATCAGAG	TCTTGGCTGAGATCCGCTGG	142	AK009918.1
Cdk4	CCAGAGATGGAGGAGTCTGG	CCTCCTTGTGCAGGTAGGAG	112	NM_001355005.1
Cdk6	TCTGGTGACCAGCAGTGGAC	TACCACAGCGTGACGACCAC	102	NM_009873.3
Ccna1	TGCCTTGCCTGAGTGAGCTG	GAACTACAGGCGGCTCCATG	126	NM_001305221.1
Ccnb2	CTGCCTCTTGCCTGTCTCAG	GCCATGTGCTGCATGACTTCC	112	NM_007630.2
Ccnd1	CTCTGTGCCACAGATGTGAAG	TTGTGCGGTAGCAGGAGAGG	127	NM_007631.2
Ccnd2	AGCTGCTGGCCAAGATCACC	TGCTGCAGGCTGTTCAGCAG	91	NM_009829.3
Ccnd3	CCATCCATGATCGCCACAGG	GATCTGTTCCTGGCAGGCTC	144	NM_001081636.1
Ccne1	TCTCCTCACTGGAGTTGATGC	CCGGAAGTGCTTGAGCTTGG	131	NM_007633.2
E2f1	AAGCAGTATTGCCACGGATGG	CTCCAGGAGTGAGTCAGCAG	89	NM_007891.5
Mdm2	GACGAGAGTGTGGAATCTAGC	AGGTGTCCAGTCTTGCCGTG	107	NM_001288586.2
Metap2	GCGTGGTTCATGACGACATGG	CACCTTCGGCAGAAGGCAAG	142	NM_019648.3
p16	CTTCCTGGACACGCTGGTG	TCGCACGATGTCTTGATGTCC	118	NM_009877.2
p21	GTGGCCTTGTCGCTGTCTTG	ATCTGTCAGGCTGGTCTGCC	108	NM_007669.5
p53	CCGGCTCTGAGTATACCACC	CCACTGGAGTCTTCCAGTGTG	112	NM_011640.3
Rb1	TTCAGAAGGTCTGCCAACACC	CCATCTGCTTCATCGGCTCC	216	NM_009029.3

© 2020 by the authors. Submitted for possible open access publication under the terms and conditions of the Creative Commons Attribution (CC BY) license (http://creativecommons.org/licenses/by/4.0/).
